# Risk of malignancy in kidney transplant recipients: a nationwide population-based cohort study

**DOI:** 10.1186/s12882-022-02796-6

**Published:** 2022-04-28

**Authors:** Su Woong Jung, Hyemi Lee, Jae Myung Cha

**Affiliations:** 1 Division of Nephrology, Department of Internal Medicine, Kyung Hee University Hospital at Gangdong, Seoul, Republic of Korea; 2 Department of Bigdata and Bioinformatics, Kyung Hee University Hospital at Gangdong, Seoul, Republic of Korea

**Keywords:** Post-transplant malignancy,, Kidney transplantation,, Relative risk,, Age,, Sex

## Abstract

**Background:**

Post-transplant malignancy is major morbidity complicated in kidney transplantation (KT). In Korea, a few studies have investigated the sex- and age-dependent risk for post-transplant malignancy among KT recipients on a large scale.

**Methods:**

We utilized a national health insurance database in Korea to investigate the relative risk of post-transplant malignancy in 12,634 KT recipients between 2007 and 2017. The same number of patients with acute appendicitis was included as a control group. The relative risk of malignancy was estimated using a multivariable-adjusted Cox model, and interaction analysis was performed to investigate age- and sex-predominant patterns.

**Results:**

KT recipients had an overall 1.8-fold higher risk for post-transplant malignancy with an increased risk for 14 of 29 cancer types, among which Kaposi’s sarcoma, non-Hodgkin’s lymphoma, kidney, uterus, and bladder/urinary tract cancers were most prominent. Although the overall risk for post-transplant malignancy was similar between male and female KT recipients, head and neck cancer had a higher risk among male KT recipients, whereas non-Hodgkin’s lymphoma and bladder/urinary tract cancer had a higher risk among female KT recipients. Overall, the young (< 50 years) KT recipients had a higher risk for post-transplant malignancy than older ones (≥ 50 years), whose pattern was most prominent in non-Hodgkin’s lymphoma. In contrast, breast and nonmelanoma skin cancer showed a higher risk among older KT recipients.

**Conclusion:**

KT recipients had an increased risk for a wide range of cancer types, some of which showed differential risk patterns with age and sex. Our result suggests that focused screening for predominant post-transplant malignancies may be an effective strategy for selected KT recipients.

**Supplementary Information:**

The online version contains supplementary material available at 10.1186/s12882-022-02796-6.

## Introduction

Patients with severely impaired renal function ultimately require renal replacement therapy, among which kidney transplantation (KT) provides superior survival benefit and better quality of life than dialysis [1, 2]. Over the past few decades, graft survival after KT has improved with the introduction of immunosuppressive drugs such as cyclosporine, tacrolimus, and mycophenolate mofetil [3, 4], and patient survival after KT has improved with decreased post-transplant cardiovascular death [5, 6]. However, the occurrence of malignancy after KT remains a major concern to overcome in post-transplant management. Population-based studies in Australia, New Zealand, and Spain demonstrated that the death rate due to post-transplant malignancy after the first KT year has surpassed the cardiovascular death rate as a result of a relatively greater reduction in cardiovascular death [5, 7]. Exact knowledge of post-transplant malignancies may help to improve patient survival after KT and guide the development of an optimal screening and surveillance plan for post-transplant malignancies.

KT recipients have an overall 2- to fourfold increased risk of post-transplant malignancy compared to the general population [8–10]. In particular, the incidence and type of post-transplant malignancy differ considerably depending on geographic location and ethnicity. For example, non-melanoma skin cancer is the most common in Western populations and comprises more than half of post-kidney transplant malignancies [11–13], whereas non-Hodgkin lymphoma is the most common in Hong Kong [14] and kidney cancer is the most frequent in Taiwan and Japan [15, 16]. Previous Korean studies on post-kidney transplant malignancies were limited by either undersampling or a relatively short follow-up period [17–19]. In this study, using the 10-year nationwide claims database in Korea, we explored the relative risk of post-kidney transplant malignancies with the comparison to the healthy general population randomly sampled from subjects undergoing acute appendicitis.

## Patients and methods

### Data source

This study was a retrospective nationwide population-based study based on Health Insurance Review and Assessment (HIRA) data, which contains all inpatient and outpatient data since 1989 in Korea. South Korea has a compulsory national health reimbursement system, which covers approximately 97% of the population and is operated under the law of fee-for-service. HIRA reviews all claims, which contain information on personal demographics, diagnostic codes, and procedures, to reimburse health care services claimed by physicians [20]. Since all personal information used in the HIRA database was encrypted into scrambled numbers before data processing, deidentified HIRA data were obtained and the requirement of informed consent was therefore waived.

### Study population

We identified KT recipients using KT procedure code (R3280) of the International Classification of Diseases, 10th revision (ICD-10) claimed between January 1, 2007, and December 31, 2017, given that procedure codes in physician claim databases almost exactly matched with data in medical charts in a previous study [21]. The following individuals were excluded from the analysis: 1) those whose KT was not the first time, 2) those who were diagnosed with any type of malignancy before KT, and 3) those who had multi-organ transplantation.

As a proxy for the general population, the same number of control population was randomly selected from individuals who had acute appendicitis (ICD-10 procedure code K35) and had not received KT during the same study period. Individuals who were diagnosed with any type of malignancy before enrollment were also excluded from the control group. The subjects in both groups were observed until the event of cancer or the study endpoint (December 31, 2018).

### Study variable

The main outcome in this study was the relative risk, which is a ratio of risk for incident cancer in KT recipients to cancer risk in control group. Since relative risk is not a raw outcome, we also presented the number of incident cases as an ancillary outcome. In our analysis, we only counted the first diagnosis of specific cancer as an event because the second diagnosis of the same cancer type probably indicates recurrence. In the case of a subject who was diagnosed with two or more cancer types, we counted all and analyzed each cancer type independently. Subgroup analyses were conducted according to sex and age groups.

Site-specific cancers were identified on the basis of ICD-10 codes according to anatomic site: head and neck, digestive, lung, connective tissue and skin, reproductive and genital tract, urinary tract, brain, thyroid, hematopoietic system, and unknown primary site (Supplementary Table 1). Cancers arising in the breast and female genital tracts were investigated only in females, whereas prostate and testicular cancers were analyzed only in males. In this study, non-melanoma skin cancers were defined as a composite of Merkel, squamous, and basal cell carcinomas. Because the Korean government implements a national insurance program that pays 95% of medical expenses for malignancy since 2005, all patients diagnosed with malignancy are registered into national database at the time of their diagnosis. Thus, the diagnostic codes for malignancy are reliable and are considered accurate epidemiologic data representing real-world situations in Korea [22, 23].

Baseline clinical characteristics collected at the time of KT (or diagnosis of acute appendicitis) included sociodemographic information, such as age, sex, socioeconomic status (represented by the insurance type), as well as comorbidities (diabetes mellitus, ischemic heart disease, heart failure, liver cirrhosis, and chronic obstructive pulmonary disease). Age groups were classified into young (< 50 years) and older (≥ 50 years) groups. Underlying comorbidities were identified using ICD-10 codes (Supplementary Table 1) and the presence of each comorbidity was defined as any relevant diagnostic code within the preceding year of enrollment.

### Statistical analysis

Baseline characteristics were displayed as numbers (percentages), and comparisons between the study groups were performed using the chi-square test. Cox proportional hazards analyses were performed to determine relative risk and its 95% confidence intervals (CIs) for malignancy in KT recipients. In Cox model, the subject was censored at the time of the first diagnosis of specific cancer. We found that proportional hazards assumption was satisfied using a plot of the log cumulative hazard which showed the parallel curves of the two groups. Multivariable models were constructed with adjustment for age (as a continuous variable), sex, and the presence of any of investigated comorbidities. We simplified comorbidity burden into the presence or absence of any comorbidities among diabetes mellitus, ischemic heart disease, heart failure, liver cirrhosis, and chronic obstructive pulmonary disease. This processed variable was incorporated as covariates instead of putting each comorbidity individually into Cox regression model to avoid unnecessary over-adjustment. Subgroup analyses were conducted according to sex and age groups. The interaction of sex (age group) and study group was additionally included as a covariate in Cox models to investigate the association of these factors. Firth’s penalized likelihood method was applied to the Cox regression model to estimate the relative risk for the cancer type that had no occurrence in the control group. All statistical analyses were conducted using SAS Enterprise Guide version 6.1 (SAS Institute, Cary, NC) or R 3.0.2 (R Foundation for Statistical Computing, Vienna, Austria). A two-sided *P* value of < 0.05 was considered statistically significant.

## Results

### Characteristics of the study population

Using a nationwide health insurance database from 2007 to 2017, a total of 12,634 KT recipients were included in this study with the same number of people diagnosed with acute appendicitis as a control group. Because acute appendicitis is a disease that usually occurs in relatively young people who have good health status [24], KT recipients were older and had more comorbidities compared to the control group. Specifically, 53% of KT recipients were over 50 years old, whereas more than two-thirds of acute appendicitis occurred in people under 50 years old (Table 1). In addition, KT recipients had a greater prevalence in all of the investigated comorbidities (diabetes, ischemic heart disease, heart failure, chronic obstructive pulmonary disease, and liver cirrhosis) than the control group. Besides, compared to the control group, the proportion of males was greater in the KT group, while the percentage of medical aid, indicative of low economic status, was also higher in KT recipients.

**Table 1:** Clinical characteristics of the study population

Clinical characteristic	KT group (*n* = 12,634)	Control group (*n* = 12,634)	*P*
Age (years)			< 0.001
Under 20	0 (0.0)	6 (0.1)	
20–34	790 (6.3)	5818 (46.1)	
35–49	5145 (40.7)	3931 (31.1)	
50–64	6040 (47.8)	1812 (14.3)	
65 or over	659 (5.2)	1067 (8.4)	
Sex			< 0.001
Male	8310 (65.8)	6073 (48.1)	
Female	4324 (34.2)	6561 (51.9)	
Insurance type			< 0.001
Social health insurance	10,781 (85.3)	12,134 (96.0)	
Medical aid	1852 (14.7)	495 (3.9)	
Veterans	1 (0.0)	5 (0.1)	
Person with comorbidities	5816 (46.0)	803 (6.4)	< 0.001
Type of comorbidities			
Diabetes mellitus	4009 (31.7)	313 (2.5)	< 0.001
Ischemic heart disease	1869 (14.8)	202 (1.6)	< 0.001
Heart failure	1241 (9.8)	65 (0.5)	< 0.001
Liver cirrhosis	1083 (8.6)	341 (2.7)	< 0.001
Chronic obstructive pulmonary disease	234 (1.9)	55 (0.4)	< 0.001

*KT* kidney transplantation

Data are presented as n (%)

### Post-kidney transplantation malignancies

A total of 1087 malignancies occurred in KT recipients, which was higher than the 864 events in the control group (*P* < 0.001). The most frequently diagnosed malignancies were stomach cancer (127 vs. 119), thyroid cancer (120 vs. 125), and colorectal cancer (72 vs. 111) in KT recipients and the control group, respectively (Supplementary Table 2). The overall risk for malignancies was significantly higher in KT recipients than in the control group (adjusted relative risk[aRR], 1.8; 95% CI, 1.6–2.0; *P* < 0.001). In detail, the risk was significantly higher in the KT recipients with the following malignancies (sites) in the order of risk: Kaposi’s sarcoma (aRR, 23.6; 95% CI, 1.3–416.8), kidney (aRR, 14.9; 95% CI, 7.9–28.3), uterus (aRR, 7.5; 95% CI, 2.0–27.6), non-Hodgkin’s lymphoma (aRR, 4.2; 95% CI, 2.7–6.6), bladder/urinary tract (aRR, 4.1; 95% CI, 2.3–7.3), non-melanoma skin (aRR, 3.7; 95% CI, 2.0–7.2), head and neck (aRR, 3.6; 95% CI, 1.5–9.1), gallbladder and bile duct (aRR, 2.5; 95% CI, 1.2–5.5), unknown primary (aRR, 2.4; 95% CI, 1.4–4.4), breast (aRR, 2.3; 95% CI, 1.5–3.5), thyroid (aRR, 2.2; 95% CI, 1.6–3.0), pancreas (aRR, 2.1; 95% CI, 1.1–4.3), stomach (aRR, 1.7, 95% CI, 1.3–2.3), and lung (aRR, 1.6; 95% CI, 1.1–2.4) (Table 2).

**Table 2:** Risk of malignancies in kidney transplantation recipients

Malignancy site	Univariable analysis		Multivariable analysis	
	Crude RR (95% CI)	*P*	Adjusted RR (95% CI)	*P*
Head and neck	3.8 (1.7–8.7)	0.002	3.6 (1.5–9.1)	0.006
Lip	1.1 (0.1–12.5)	0.926	3.9 (0.3–50.0)	0.295
Digestive				
Esophagus	1.6 (0.5–4.9)	0.391	1.7 (0.5–6.6)	0.415
Stomach	1.7 (1.3–2.2)	< 0.001	1.7 (1.3–2.3)	< 0.001
Colon and rectum	1.0 (0.7–1.3)	0.893	1.0 (0.7–1.3)	0.776
Anus	8.9 (0.2–365.2)	0.250	16.4 (0.4–682.9)	0.142
Liver	1.8 (1.3–22.5)	0.001	1.3 (0.9–1.8)	0.224
Gallbladder and bile duct	1.6 (0.8–3.2)	0.189	2.5 (1.2–5.5)	0.021
Pancreas	2.3 (1.2–4.2)	0.011	2.1 (1.1–4.3)	0.032
Lung	1.8 (1.3–2.5)	< 0.001	1.6 (1.1–2.4)	0.010
Bone and cartilage	1.4 (0.2–10.4)	0.735	4.3 (0.2–95.3)	0.356
Melanoma	2.3 (0.4–11.9)	0.321	0.9 (0.1–5.8)	0.919
Non-melanoma skin cancer^a^	5.2 (2.9–9.4)	< 0.001	3.7 (2.0–7.2)	< 0.001
Kaposi’s sarcoma	32.1 (1.7–588.4)	0.020	23.6 (1.3–416.8)	0.031
Breast	1.4 (1.0–1.9)	0.078	2.3 (1.5–3.5)	< 0.001
Uterine cervix	0.5 (0.2–1.1)	0.090	0.7 (0.3–1.6)	0.375
Uterus	2.6 (1.0–6.6)	0.053	7.5 (2.0–27.6)	0.003
Ovary	0.9 (0.4–1.8)	0.673	1.9 (0.8–4.8)	0.154
Vulva, vagina	0.3 (0.0–14.7)	0.568	0.5 (0.0–41.1)	0.742
Prostate	1.3 (0.9–1.9)	0.154	1.0 (0.6–1.5)	0.892
Testis	4.1 (0.5–36.8)	0.207	2.8 (0.3–28.2)	0.377
Urinary tract				
Kidney	14.8 (7.9–27.4)	< 0.001	14.9 (7.9–28.3)	< 0.001
Bladder/urinary tract	4.4 (2.6–7.5)	< 0.001	4.1 (2.3–7.3)	< 0.001
Brain	1.0 (0.4–2.3)	0.943	1.3 (0.5–3.2)	0.620
Thyroid	1.4 (1.1–1.8)	0.007	2.2 (1.6–3.0)	< 0.001
Hematopoietic system				
Hodgkin’s lymphoma	1.4 (0.2–9.9)	0.761	2.7 (0.3–22.5)	0.348
NHL	3.4 (2.2–5.1)	< 0.001	4.2 (2.7–6.6)	< 0.001
Multiple myeloma	2.8 (0.9–9.3)	0.087	1.6 (0.4–6.2)	0.491
Leukemia	1.1 (0.5–2.4)	0.824	0.7 (0.3–1.8)	0.514
Unknown primary	2.6 (1.5–4.4)	< 0.001	2.4 (1.4–4.4)	0.003
Total	1.9 (1.7–2.1)	< 0.001	1.8 (1.6–2.0)	< 0.001

*KT* kidney transplantation, *CI* confidence interval, *RR* relative risk, *NHL* Non–Hodgkin’s lymphoma

The relative risk was obtained from the Cox model adjusted for age, sex, and the presence or absence of any of investigated comorbidities as in Table 1

^a^Non-melanoma skin cancer is a composite of Merkel, squamous, and basal cell carcinomas

^b^Cancers of the breast and female genital tracts were investigated only in females, whereas prostate and testicular cancers were analyzed only in males

### Post-kidney transplantation malignancy according to sex

The total cancer occurrences per 10,000 male subjects were 882 vs. 650 in KT recipients and control groups, whose predominant cancer types showed different patterns; the top most common cancers were kidney, stomach, and liver cancers in KT recipients and stomach, prostate, and colorectal cancers in control group in the order of incident cases (Supplementary Table 2). In the case of females, a total of 819 vs. 715 cancers were newly diagnosed per 10,000 KT recipient and control subjects, among which thyroid, breast, and stomach cancers comprise the most common cancer in both groups.

**Fig. 1:**
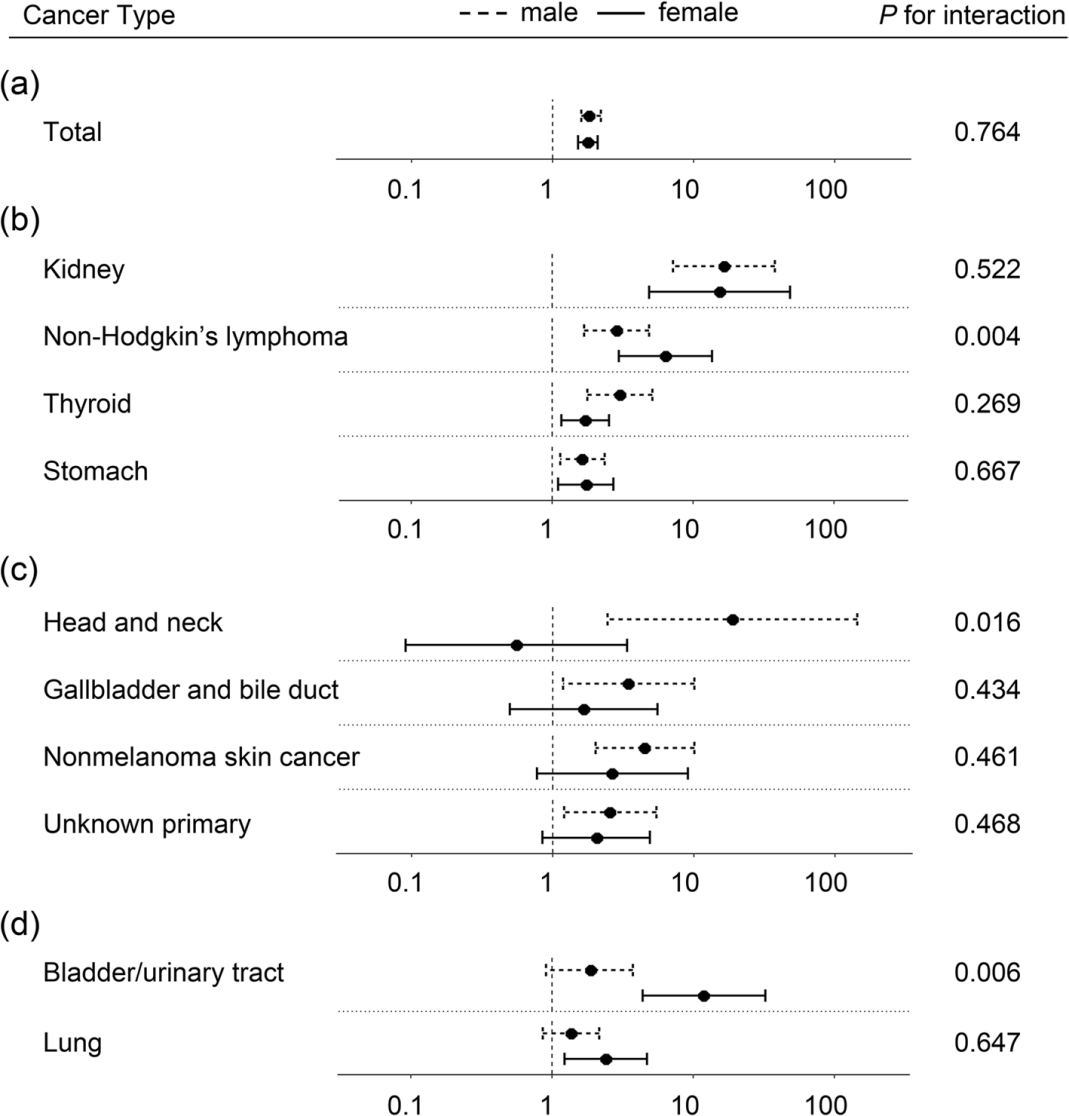
Forest plot presenting the relative risk for post-transplant malignancy according to sex. The forest plot was depicted for the composite of cancers **A** and cancers whose risk was elevated in both sexes **B**, only in males **C**, or females **D**. The circles represent relative risks, and the horizontal lines indicate 95% confidence intervals. The dashed lines represent male sex, while the solid lines denote female sex. The relative risk was obtained from the Cox regression model adjusted for age, sex, and the presence or absence of any of investigated comorbidities as in Table 1

Overall, the relative risk for malignancy did not differ between male and female KT recipients (Fig. 1a and Supplementary Table 3). In both sexes, KT recipients had a significantly increased risk of kidney cancer, non-Hodgkin’s lymphoma, thyroid, and stomach cancers compared to the control group (Fig. 1b). The relative risk was specifically increased in male KT recipients for head and neck, gallbladder and bile duct, non-melanoma skin, and unknown primary cancers with greater than twofold excess (Fig. 1c), whereas the relative risk for bladder/urinary tract and lung cancers was only significant in females (Fig. 1d). Of those, the interaction between sex (male vs. female) and the study group (KT vs. control group) was statistically significant for head and neck cancer (*P* = 0.016) in males and non-Hodgkin’s lymphoma (*P* = 0.004) and bladder/urinary tract cancer (*P* = 0.006) in females.

### Post-kidney transplantation malignancy according to age

Observed events of nearly all cancer types were more common in older people (≥ 50 years). Specifically, a total of 463 vs. 1212 cancers were newly diagnosed per 10,000 young and older KT recipients, while 323 vs. 1907 cases occurred per 10,000 young and older control subjects. Kidney cancer, thyroid cancer, and non-Hodgkin’s lymphoma were the most frequent cancers among young KT recipients (< 50 years), while stomach, kidney, and thyroid cancers were the most common cancers among older ones in the order of incidence (Supplementary Table 2).

**Fig. 2:**
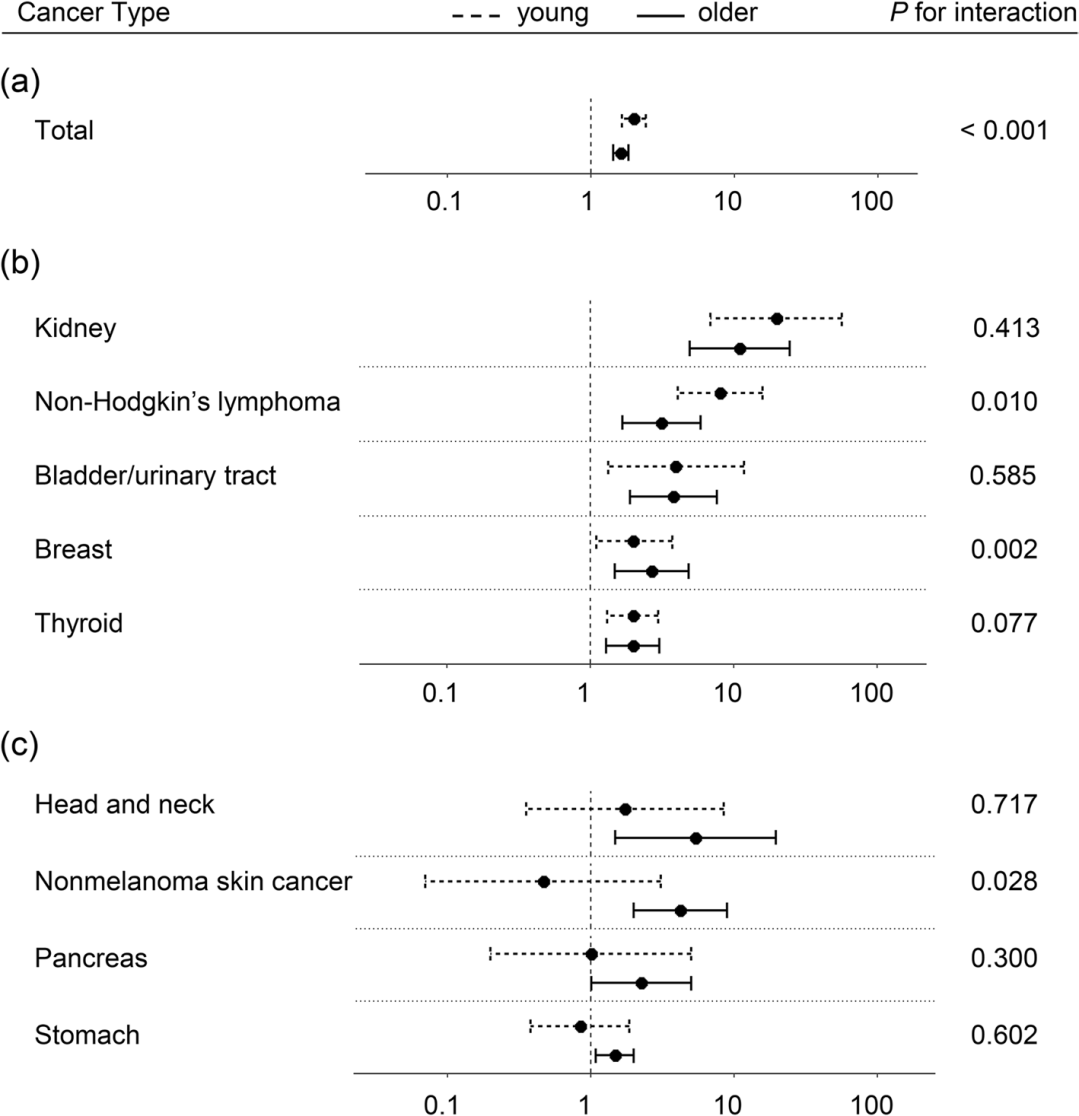
Forest plot presenting the relative risk for post-transplant malignancy according to age group.The forest plot was depicted for the composite of cancers **A** and cancers whose risk was elevated in both young (< 50) and older (≥ 50) people **B** or only in older ones **C**. The circles represent relative risks, and the horizontal lines indicate 95% confidence intervals. The dashed lines represent the young, while the solid lines denote the older. The relative risk was obtained from the Cox regression model adjusted for age, sex, and the presence or absence of any of investigated comorbidities as in Table 1

Despite lower incident cases in young KT recipients, young people showed a relatively higher risk for post-transplant malignancies than older people (aRR, 1.99 vs. 1.63) (Fig. 2a and Supplementary Table 4). This pattern was statistically significant in the interaction analysis between age (young vs. older) and the study group (KT vs. control group) (*P* < 0.001, Fig. 2a), which was largely attributed to a much greater risk for kidney cancer (aRR, 20.0 vs. 11.1) and non-Hodgkin’s lymphoma (aRR, 8.1 vs. 3.2) in young KT recipients (Fig. 2b).

In both young and older groups, an approximately fourfold higher relative risk was noted for bladder/urinary tract cancer, and an approximately twofold higher relative risk was observed for breast and thyroid cancers. In particular, older KT recipients had elevated relative risk for head and neck (5.4-fold), non-melanoma skin (4.2-fold), pancreas (2.3-fold), and stomach (50%) cancers in contrast to the non-significant risk in young KT recipients (Fig. 2c).

In the interaction analysis between age (young vs. older) and the study group (KT vs. control group), non-Hodgkin’s lymphoma (*P* = 0.010) had a higher risk among young KT recipients, while breast (*P* = 0.002) and nonmelanoma skin cancers (*P* = 0.028) had higher risk among older KT recipients.

### Time to cancer diagnosis in the study population

The interval time to cancer diagnosis in KT recipients was shorter than that in the control group for all cancer types except for testicular and brain cancers. The median time to composite cancer diagnosis was 2.9 and 5.0 years in the KT recipients and control groups, respectively (Table 3). Of the cancer types whose cases were more than 10 in both study groups, KT recipients had an earlier diagnosis of ≥ 2 years in ovarian (4.9 years earlier), kidney (3.7 years earlier), breast (2.8 years earlier), thyroid (2.8 years earlier), and liver (2.0 years earlier) cancers compared to the control group.

**Table 3:** Time to malignancy diagnosis in the study population

Malignancy	KT group (*n* = 12,634)	Control group (*n* = 12,634)
Head and neck	2.97 (1.81–4.75)	6.70 (3.96–7.94)
Lip	5.12 (5.12 5.12)	6.36 (4.91–7.81)
Digestive		
Esophagus	2.45 (0.57–4.79)	4.49 (3.97–6.30)
Stomach	3.89 (2.23–5.19)	5.53 (2.93–7.41)
Colon and rectum	3.87 (2.01–6.13)	4.26 (1.48–6.88)
Anus	5.31 (5.10–5.52)	NA
Liver	3.11 (1.31–4.63)	5.16 (2.25–7.52)
Gallbladder and bile duct	3.97 (1.03–4.88)	4.21 (3.70–4.86)
Pancreas	1.93 (0.68–4.50)	3.93 (2.94–8.53)
Lung	3.99 (1.55–5.31)	5.19 (3.01–7.07)
Connective tissue and skin		
Bone and cartilage	2.92 (0.00–5.85)	4.38 (1.76–7.01)
Melanoma	6.02 (2.82–9.03)	9.81 (3.51–9.82)
Non-melanoma skin cancer	4.58 (2.88–6.95)	5.19 (3.22–7.11)
Kaposi’s sarcoma	1.12 (0.84–1.80)	NA
Reproductive and genital tract		
Breast	2.63 (1.53–6.71)	5.47 (4.33–8.22)
Uterine cervix	3.02 (1.42–5.41)	5.17 (1.45–7.00)
Uterus	2.72 (0.65–3.72)	7.23 (4.04–7.88)
Ovary	0.85 (0.51–4.03)	5.81 (1.93–7.85)
Vulva, vagina	NA	5.23 (2.72–7.73)
Prostate	2.95 (1.25–5.31)	4.05 (0.00–6.52)
Testis	1.59 (0.41–2.49)	0.84 (0.84–0.84)
Urinary tract		
Kidney	1.10 (0.16–3.37)	4.82 (0.45–7.10)
Bladder/urinary tract	3.00 (1.69–5.70)	3.73 (2.87–6.49)
Brain	4.05 (1.56–4.13)	2.83 (1.78–4.25)
Thyroid	2.32 (0.90–4.33)	5.15 (3.29–7.21)
Hematopoietic system		
Hodgkin’s lymphoma	1.22 (0.85–1.59)	5.40 (5.23–5.56)
NHL	4.14 (1.54–5.68)	4.56 (2.47–7.78)
Multiple myeloma	1.81 (0.93–1.96)	3.81 (1.20–6.51)
Leukemia	3.30 (2.00–5.85)	4.73 (3.39–7.99)
Unknown primary	3.87 (1.97–5.56)	4.39 (2.18–6.52)
Total	2.94 (1.19–5.11)	4.96 (2.70–7.32)

*KT* kidney transplantation, *NA* not applicable, *NHL* Non–Hodgkin’s lymphoma

Data are presented as median (interquartile range), and interval periods to cancer diagnosis were calculated in years

## Discussion

This nationwide study provides a landscape of cancer-type-specific risk after KT with comparison to the control group based on a population database of over 12,000 Korean KT recipients. Overall, KT recipients had a 1.8-fold higher risk for post-transplant malignancies in this study. This risk was similar in male and female KT recipients but differed by age, with a higher risk in young people.

The incidence of each type of cancer has geographic and racial variations. In Korea, the top five most common cancers are stomach, colorectal, thyroid, lung, and breast cancers [25]. The control group in this study demonstrated this pattern, indicating adequate sampling representative of the general population. In contrast, KT recipients have different profiles of cancer development largely due to immunosuppression and impaired immunosurveillance against oncogenic virus. In this study, the greatest risk was observed with Kaposi’s sarcoma, whose etiology is human herpesvirus 8, whereas none of the control groups developed Kaposi’s sarcoma. In addition, the two cases of anal cancer, most of which are developed by human papillomavirus infection, were seen only in KT recipients. The incidence of non-Hodgkin’s lymphoma, Epstein-Barr virus-related malignancy, was also elevated by a factor of 4.5 times in KT recipients.

Korea is one of the most prevalent areas in the world for stomach cancer [26], with the risk for this malignancy being 1.7-times higher in KT recipients in this study. The reason for this observation may be speculated to enhanced Helicobacter pylori infection under immunosuppression, but this association is neither certain nor proven [27–29].

Lip cancer in East Asia is not as prevalent as in Western countries [30]. In Western studies, lip cancer accounted for two-thirds or more cases of oral cavity cancer in KT recipients [8, 9, 12], while only one and two cases of lip cancer were detected among KT recipients and control group in this study, respectively.

KT recipients were at increased risk for kidney, bladder/urinary tract, and thyroid cancers as well. These cancers were previously categorized as end-stage renal disease (ESRD)-related cancers since their risk was already elevated during the dialysis period before KT [8, 31] and were not increased in other immunosuppressive states, such as human immunodeficiency virus infection (30). A higher incidence of cancer arising in the urinary system is likely to be an intrinsic factor to this anatomical region associated with ESRD, rather than solely due to immunosuppression. Interestingly, bladder/urinary tract cancer in KT recipients showed female predominance in contrast to male predominance in the general population [32]. This epidemiologic feature was also evident in previous studies conducted in Taiwan and Hong Kong [14, 16, 33], and our study validated this finding again by interaction analysis. The higher incidence of thyroid cancer in KT recipients may be explained by the lingering effect of precedent uremic milieu, although its risk was alleviated after KT compared to the dialysis period [34]. Our study showed a high incidence of thyroid cancer in both groups, and this phenomenon is thought to be a result of widespread and easily accessible screening for thyroid cancer in Korea [35].

Aging is a well-established risk factor for malignancy; however, when compared to the general population, the relative risk for post-transplant malignancy was higher in young KT recipients as evidenced by the statistical significance of the interaction analysis. Our finding is consistent with those of Canadian, Taiwanese, and Australian studies [9, 16, 31]. Contrary to this overall pattern, older people had a significantly higher risk of non-melanoma skin cancers than young people. Squamous and basal cell carcinomas comprise the vast majority of non-melanoma skin cancers [12, 36], and accumulated prior exposure to ultraviolet light possibly accounts for this older preponderance. The incidence of head and neck cancers was also much more pronounced in older people with a male-predominant pattern, which could be confounded by habitual status such as smoking.

This study had several limitations. First, the two study groups were not balanced in terms of baseline demographic and comorbidity status. Although we adjusted for age, sex, and comorbidities in Cox proportional hazard model, a portion of relative risk could be derived from the residual effect of unbalanced covariables given their probable positive associations with some cancer types [37, 38]. Nonetheless, we believe that our data genuinely represents the real world that a physician commonly encounters in clinical practice. Second, some of the cancers may be unrelated to KT since the events of malignancy were counted right after KT without a washout period to obtain incidence under the same condition in the control group. Third, detailed demographic data, such as smoking and alcohol use, as well as clinical information on donor types, immunosuppressive medications, histologic types, and stages of cancer were not available in the HIRA database, and we were thus unable to consider the impact of those factors on the relative risk for post-transplant malignancies. Lastly, the relative risk derived from Cox model was not death- or graft loss-censored. Thus, ignoring potential competing risks possibly overestimates the risk for post-transplant malignancies, but the overall relative risk in this study (1.8) was lower than previously reported results (2.5 to 3.8) calculated as a standardized incidence ratio [9, 14, 16].

In conclusion, this study reaffirmed a higher risk of malignancy among KT recipients for 14 of 29 cancer types, most of which were diagnosed earlier than the control group. Despite the life-threatening impact of post-transplant malignancies, cancer screening in KT recipients generally follows the guidelines for the general population because of the lack of well-designed randomized controlled trials in this at-risk population [39, 40]. Given differential risk patterns in some cancers according to age and sex, our results suggest that focused and more thorough screening for those cancer types may be warranted for a subset of KT recipients.

## Conclusion

This study highlights the differential risks for post-transplant malignancies in Korean KT recipients depending on age and sex. For example, head and neck cancer showed a higher risk in male KT recipients, whereas non-Hodgkin’s lymphoma and bladder/urinary tract cancer had a higher risk in female ones. Although the overall risk for post-transplant malignancy was higher among young KT recipients despite its lower incidence compared to older ones, the relative risk for breast and nonmelanoma skin cancers was higher among older ones. Our result suggests that focused screening for predominant post-transplant malignancies may be an effective strategy among a subset of KT recipients who have particularly at higher risk.

## Supplementary Information

**Additional file 1.** 

## Data Availability

The datasets used and/or analysed during the current study available from the corresponding author on reasonable request.

## Abbreviations

aRR: Adjusted Relative Risk
CI: Confidence Interval
COPD: Chronic Obstructive Pulmonary Disease
ESRD: End-stage Renal Disease
HIRA: Health Insurance Review and Assessment
KT: Kidney Transplantation
NA: Not Applicable
NHL: Non–Hodgkin’s Lymphoma
