# Supplementary material for: Risk of malignancy in kidney transplant recipients: a nationwide population-based cohort study
Source: BMC Nephrol. 2022 Apr 28;23:160. doi: 10.1186/s12882-022-02796-6 (PMC9047256; doi:10.1186/s12882-022-02796-6)
Supplement: Supplementary file 1 — Additional file 1. [file 12882_2022_2796_MOESM1_ESM.docx]

**Supplementary Information
Additional file 1: Supplementary Table 1.** ICD-10 diagnostic codes for internal malignancies and comorbidities
**Additional file 1: Supplementary Table 2.** Incident case of malignancy in the study population **Additional file 1: Supplementary Table 3.** Relative risk for malignancies in kidney transplantation recipients according to sex **Additional file 1: Supplementary Table 4.** Relative risk for malignancies in kidney transplantation recipients according to age groups

**Supplementary Table 1. ICD–10 diagnostic codes for internal malignancies and comorbidities**

| ICD–10 codes for malignancy | ICD–10 codes |  | ICD–10 codes for comorbidities | ICD–10 codes |
| --- | --- | --- | --- | --- |
| Head and neck | C00–C14, C30–C32 |  | Diabetes mellitus | E10–E14 |
| Lip | C00 |  | Ischemic heart disease | I20–I25 |
| Digestive |  |  | Heart failure | I110, I130, I132, I50 |
| Esophagus | C15 |  | Liver cirrhosis | K703, K74, K76 |
| Stomach | C16 |  | Chronic obstructive pulmonary disease | J44 |
| Colon and rectum | C18–C20 |  |  |  |
| Anus | C21 |  |  |  |
| Liver | C22 |  |  |  |
| Gallbladder and bile duct | C23–C24 |  |  |  |
| Pancreas | C25 |  |  |  |
| Lung | C34 |  |  |  |
| Connective tissue and skin |  |  |  |  |
| Bone and cartilage | C40–C41 |  |  |  |
| Melanoma | C43, D03 |  |  |  |
| Non-melanoma skin cancer | C44, C4A |  |  |  |
| Kaposi’s sarcoma | C46 |  |  |  |
| Reproductive and genital tract |  |  |  |  |
| Breast | C50 |  |  |  |
| Uterine cervix | C53 |  |  |  |
| Uterus | C54–C55 |  |  |  |
| Ovary | C56 |  |  |  |
| Vulva, vagina | C51–C52 |  |  |  |
| Prostate | C61 |  |  |  |
| Testis | C62 |  |  |  |
| Urinary tract |  |  |  |  |
| Kidney | C64 |  |  |  |
| Bladder/urinary tract | C65–C67 |  |  |  |
| Brain | C71 |  |  |  |
| Thyroid | C73 |  |  |  |
| Hematopoietic system |  |  |  |  |
| Hodgkin’s lymphoma | C81 |  |  |  |
| Non– Hodgkin’s lymphoma | C82–C86, C88 |  |  |  |
| Multiple myeloma | C90 |  |  |  |
| Leukemia | C91–C95 |  |  |  |
| Unknown primary | C76, C79, C80 |  |  |  |

*ICD-10* International Classification of Diseases-10th revision

**Supplementary Table 2. Incident case of malignancy in the study population**

| Group | KT group | | | | |  | Control group | | | | |
| --- | --- | --- | --- | --- | --- | --- | --- | --- | --- | --- | --- |
|  | Total | Male | Female | Young^a^ | Older^a^ |  | Total | Male | Female | Young^a^ | Older^a^ |
| Number of population | 12,634 | 8,310 | 4,324 | 5,935 | 6,699 |  | 12,634 | 6,073 | 6,561 | 9,755 | 2,879 |
| Head and neck | 20 | 18 | 2 | 4 | 16 |  | 8 | 1 | 7 | 5 | 3 |
| Lip | 1 | 0 | 1 | 0 | 1 |  | 2 | 0 | 2 | 0 | 2 |
| Digestive |  |  |  |  |  |  |  |  |  |  |  |
| Esophagus | 7 | 6 | 1 | 0 | 7 |  | 6 | 6 | 0 | 1 | 5 |
| Stomach | 127 | 90 | 37 | 12 | 115 |  | 119 | 61 | 58 | 25 | 94 |
| Colon and rectum | 72 | 52 | 20 | 21 | 51 |  | 111 | 58 | 53 | 30 | 81 |
| Anus | 2 | 0 | 2 | 0 | 2 |  | 0 | 0 | 0 | 0 | 0 |
| Liver | 78 | 65 | 13 | 20 | 58 |  | 66 | 43 | 23 | 14 | 52 |
| Gallbladder and bile duct | 17 | 12 | 5 | 1 | 16 |  | 15 | 7 | 8 | 1 | 14 |
| Pancreas | 26 | 19 | 7 | 3 | 23 |  | 17 | 4 | 13 | 6 | 11 |
| Lung | 79 | 63 | 16 | 9 | 70 |  | 69 | 43 | 26 | 9 | 60 |
| Connective tissue and skin |  |  |  |  |  |  |  |  |  |  |  |
| Bone and cartilage | 2 | 1 | 1 | 1 | 1 |  | 2 | 0 | 2 | 2 | 0 |
| Melanoma | 3 | 3 | 0 | 0 | 3 |  | 3 | 1 | 2 | 1 | 2 |
| Non-melanoma skin cancer^b^ | 45 | 38 | 7 | 2 | 43 |  | 15 | 9 | 6 | 5 | 10 |
| Kaposi’s sarcoma | 15 | 13 | 2 | 6 | 9 |  | 0 | 0 | 0 | 0 | 0 |
| Reproductive and genital tract |  |  |  |  |  |  |  |  |  |  |  |
| Breast | 59 | NA | 59 | 22 | 37 |  | 73 | NA | 73 | 51 | 22 |
| Uterine cervix | 8 | NA | 8 | 1 | 7 |  | 24 | NA | 24 | 10 | 14 |
| Uterus | 12 | NA | 12 | 3 | 9 |  | 7 | NA | 7 | 5 | 2 |
| Ovary | 12 | NA | 12 | 5 | 7 |  | 20 | NA | 20 | 15 | 5 |
| Vulva, vagina | 0 | NA | 0 | 0 | 0 |  | 2 | NA | 2 | 0 | 2 |
| Prostate | 54 | 54 | NA | 7 | 47 |  | 59 | 59 | NA | 10 | 49 |
| Testis | 4 | 4 | NA | 1 | 3 |  | 1 | 1 | NA | 0 | 1 |
| Urinary tract |  |  |  |  |  |  |  |  |  |  |  |
| Kidney | 127 | 107 | 20 | 51 | 76 |  | 11 | 6 | 5 | 4 | 7 |
| Bladder/urinary tract | 55 | 31 | 24 | 13 | 42 |  | 19 | 14 | 5 | 7 | 12 |
| Brain | 9 | 7 | 2 | 2 | 7 |  | 13 | 6 | 7 | 2 | 11 |
| Thyroid | 120 | 59 | 61 | 49 | 71 |  | 125 | 22 | 103 | 81 | 44 |
| Hematopoietic system |  |  |  |  |  |  |  |  |  |  |  |
| Hodgkin’s lymphoma | 2 | 2 | 0 | 1 | 1 |  | 2 | 0 | 2 | 0 | 2 |
| Non–Hodgkin’s lymphoma | 73 | 49 | 24 | 29 | 44 |  | 34 | 24 | 10 | 18 | 16 |
| Multiple myeloma | 9 | 7 | 2 | 2 | 7 |  | 4 | 3 | 1 | 0 | 4 |
| Leukemia | 11 | 9 | 2 | 1 | 10 |  | 16 | 15 | 1 | 8 | 8 |
| Unknown primary | 39 | 24 | 15 | 9 | 30 |  | 23 | 12 | 11 | 5 | 18 |
| Total | 1087 | 733 | 354 | 275 | 812 |  | 864 | 395 | 469 | 315 | 549 |

*KT* kidney transplantation, *NA* not applicable.
^a^Young denotes the people whose age was under 50 years old, whereas older does the ones whose age was 50 or over.
^b^Non-melanoma skin cancer is a composite of Merkel, squamous, and basal cell carcinomas.

**Supplementary Table 3. Relative risk for malignancies in kidney transplantation recipients according to sex**

| Malignancy site | Male | | | | |  | Female | | | | |
| --- | --- | --- | --- | --- | --- | --- | --- | --- | --- | --- | --- |
|  | Univariable analysis | |  | Multivariable analysis | |  | Univariable analysis | |  | Multivariable analysis | |
|  | Crude HR  (95% CI) | *P* |  | Adjusted HR  (95% CI) | *P* |  | Crude HR  (95% CI) | *P* |  | Adjusted HR  (95% CI) | *P* |
| Head and neck | 17.7 (2.3–133.0) | 0.005 |  | 18.8 (2.4–145.3) | 0.005 |  | 0.8 (0.2–3.7) | 0.739 |  | 0.6 (0.1–3.4) | 0.526 |
| Lip | NA | NA |  | NA | NA |  | 1.8 (0.2–20.5) | 0.624 |  | 3.9 (0.3–50.0) | 0.295 |
| Digestive |  |  |  |  |  |  |  |  |  |  |  |
| Esophagus | 0.9 (0.3–2.9) | 0.890 |  | 1.5 (0.4–5.7) | 0.596 |  | 11.1 (0.1–1031.0) | 0.298 |  | 3.8 (0.0–588.8) | 0.601 |
| Stomach | 1.6 (1.1–2.2) | 0.006 |  | 1.6 (1.1–2.4) | 0.008 |  | 1.6 (1.0–2.4) | 0.030 |  | 1.7 (1.1–2.7) | 0.016 |
| Colon and rectum | 0.9 (0.6–1.4) | 0.682 |  | 0.9 (0.6–1.4) | 0.788 |  | 0.9 (0.5–1.5) | 0.699 |  | 0.9 (0.5–1.6) | 0.784 |
| Anus | NA | NA |  | NA | NA |  | 13.0 (0.3–536.1) | 0.176 |  | 16.5 (0.4–628.1) | 0.132 |
| Liver | 1.6 (1.1–2.4) | 0.014 |  | 1.4 (0.9–2.2) | 0.131 |  | 1.2 (0.6–2.3) | 0.661 |  | 1.0 (0.5–2.1) | 0.963 |
| Gallbladder and bile duct | 1.8 (0.7–4.6) | 0.231 |  | 3.4 (1.2–10.1) | 0.024 |  | 1.2 (0.4–3.8) | 0.717 |  | 1.7 (0.5–5.6) | 1.673 |
| Pancreas | 3.8 (1.3–11.3) | 0.015 |  | 3.2 (1.0–10.1) | 0.053 |  | 1.6 (0.6–4.0) | 0.361 |  | 1.6 (0.6–4.5) | 0.338 |
| Lung | 1.5 (1.0–2.3) | 0.032 |  | 1.4 (0.9–2.2) | 0.180 |  | 1.6 (0.9–3.0) | 0.136 |  | 2.4 (1.2–4.8) | 0.011 |
| Connective tissue and skin |  |  |  |  |  |  |  |  |  |  |  |
| Bone and cartilage | 1.8 (0.0–180.1) | 0.795 |  | 2.0 (0.0–623.9) | 0.813 |  | 1.5 (0.1–16.2) | 0.730 |  | 1.4 (0.1–25.6) | 0.841 |
| Melanoma | 3.7 (0.4–36.7) | 0.259 |  | 2.9 (0.2–35.4) | 0.394 |  | 1.2 (0.0–49.0) | 0.927 |  | 0.2 (0.0–17.9) | 0.512 |
| Non-melanoma skin cancer^*^ | 5.0 (2.4–10.3) | < 0.001 |  | 4.5 (2.0–10.1) | < 0.001 |  | 3.2 (1.1–9.6) | 0.041 |  | 2.7 (0. 8–9.1) | 0.119 |
| Kaposi’s sarcoma | 19.3 (1.0–361.3) | 0.048 |  | 17.5 (0.9–325.2) | 0.055 |  | 8.1 (0.2–333.6) | 0.272 |  | 9. 7 (0.1–703.3) | 0.300 |
| Urinary tract |  |  |  |  |  |  |  |  |  |  |  |
| Kidney | 15.1 (6.6–34.5) | < 0.001 |  | 16.7 (7.2–38.5) | < 0.001 |  | 8.9 (3.3–24.0) | < 0.001 |  | 15.4 (4.9–48.9) | < 0.001 |
| Bladder/urinary tract | 2.3 (1.2–4.3) | 0.012 |  | 1.9 (0.9–3.8) | 0.082 |  | 11.2 (4.2–29.6) | < 0.001 |  | 11.9 (4.4–32.5) | < 0.001 |
| Brain | 1.1 (0.4–3.4) | 0.813 |  | 1.4 (0.4–4.4) | 0.608 |  | 0.6 (0.1–2.9) | 0.529 |  | 1.0 (0.2–4.9) | 0.993 |
| Thyroid | 2.5 (1.5–4.2) | < 0.001 |  | 3.1 (1.8–5.2) | < 0.001 |  | 1.4 (1.0–1.9) | 0.038 |  | 1.7 (1.2–2.5) | 0.005 |
| Hematopoietic system |  |  |  |  |  |  |  |  |  |  |  |
| Hodgkin’s lymphoma | 3.5 (0.1–144.2) | 0.510 |  | 8.2 (0.1–461.6) | 0.307 |  | 0.5 (0.0–22.0) | 0.740 |  | 0.5 (0.0–27.2) | 0.756 |
| Non–Hodgkin’s lymphoma | 2.2 (1.3–3.6) | 0.002 |  | 2.9 (1.7–4.9) | < 0.001 |  | 5.3 (2.5–11.2) | <0.001 |  | 6.4 (3.0–13.8) | < 0.001 |
| Multiple myeloma | 2.0 (0.5–7.9) | 0.324 |  | 1.1 (0.2–5.2) | 0.892 |  | 4.0 (0.4–45.1) | 0.257 |  | 4.1 (0.3–52.5) | 0.279 |
| Leukemia | 0.7 (0.3–1.5) | 0.335 |  | 0.5 (0.2–1.4) | 0.196 |  | 5.2 (0.4–63.2) | 0.199 |  | 8.6 (0.6–116.2) | 0.104 |
| Unknown primary | 2.0 (1.0–4.1) | 0.050 |  | 2.6 (1.2–5.5) | 0.014 |  | 3.4 (1.5–7.5) | 0.002 |  | 2.0 (0.9–4.9) | 0.110 |
| Total | 1.9 (1.7–2.1) | < 0.001 |  | 1.8 (1.6–2.2) | < 0.001 |  | 1.8 (1.6–2.1) | <0.001 |  | 1.8 (1.5–2.1) | < 0.001 |

*HR* hazard ratio, *CI* confidence interval, *NA* not applicable.

The relative risk was obtained from the Cox model adjusted for age, sex, and the presence or absence of any of investigated comorbidities as in Table 1.

^*^Non-melanoma skin cancer is a composite of Merkel, squamous, and basal cell carcinomas.

**Supplementary Table 4. Relative risk for malignancies in kidney transplantation recipients according to age groups**

| Malignancy site | Young subjects (< 50 years) | | | | |  | Older subjects (≥ 50 years) | | | | |
| --- | --- | --- | --- | --- | --- | --- | --- | --- | --- | --- | --- |
|  | Univariable analysis | |  | Multivariable analysis | |  | Univariable analysis | |  | Multivariable analysis | |
|  | Crude HR  (95% CI) | *P* |  | Adjusted HR  (95% CI) | *P* |  | Crude HR  (95% CI) | *P* |  | Adjusted HR  (95% CI) | *P* |
| Head and neck | 2.0 (0.5–7.4) | 0.323 |  | 1.7 (0.4–8.5) | 0.493 |  | 5.6 (1.6–19.2) | 0.007 |  | 5.4 (1.5–19.8) | 0.010 |
| Lip | NA | NA |  | NA | NA |  | 0.8 (0.1–9.1) | 0.870 |  | 2.7 (0.2–31.7) | 0.432 |
| Digestive |  |  |  |  |  |  |  |  |  |  |  |
| Esophagus | 0.8 (0.0–74.5) | 0.907 |  | 0.4 (0.0–62.4) | 0.702 |  | 1.4 (0.4–4.4) | 0.596 |  | 1.0 (0.3–3.8) | 0.962 |
| Stomach | 1.2 (0.6–2.4) | 0.603 |  | 0.8 (0.4–1.9) | 0.683 |  | 1.4 (1.0–1.8) | 0.022 |  | 1.5 (1.1–2.0) | 0.012 |
| Colon and rectum | 1.6 (0.9–2.9) | 0.901 |  | 1.3 (0.7–2.5) | 0.365 |  | 0.7 (0.5–1.0) | 0.0312 |  | 0.7 (0.5–1.1) | 0.088 |
| Anus | NA | NA |  | NA | NA |  | 6.4 (0.2–262.0) | 0.330 |  | 9.8 (0.2–501.1) | 0.256 |
| Liver | 3.1 (1.5–6.1) | 0.002 |  | 1.8 (0.8–3.8) | 0.140 |  | 1.3 (0.9–1.8) | 0.253 |  | 0.9 (0.6–1.5) | 0.796 |
| Gallbladder and bile duct | 1.9 (0.1–30.7) | 0.657 |  | 1.7 (0.1–27.3) | 0.726 |  | 1.2 (0.6–2.4) | 0.681 |  | 1.5 (0.7–3.3) | 0.277 |
| Pancreas | 1.0 (0.3–4.2) | 0.946 |  | 1.0 (0.2–5.0) | 0.994 |  | 2.4 (1.2–5.0) | 0.020 |  | 2.3 (1.0–5.1) | 0.047 |
| Lung | 2.1 (0.8–5.4) | 0.118 |  | 1.4 (0.5–4.2) | 0.528 |  | 1.4 (1.0–2.0) | 0.074 |  | 1.1 (0.8–1.7) | 0.539 |
| Connective tissue and skin |  |  |  |  |  |  |  |  |  |  |  |
| Bone and cartilage | 1.1 (0.1–12.1) | 0.933 |  | 1.4 (0.1–38.4) | 0.845 |  | 4.0 (0.0–366.1) | 0.545 |  | 15.0 (0.1–3977.6) | 0.342 |
| Melanoma | 0.7 (0.0–69.6) | 0.884 |  | 0.5 (0.1–3.1) | 0.685 |  | 3.6 (0.5–23.9) | 0.189 |  | 0.9 (0.1–9.5) | 0.922 |
| Non-melanoma skin cancer^*^ | 0.9 (0.2–4.7) | 0.885 |  | 0.5 (0.1–3.1) | 0.467 |  | 5.5 (2.7–11.0) | < 0.001 |  | 4.2 (2.0–8.9) | < 0.001 |
| Kaposi’s sarcoma | 20.9 (0.9–469.0) | 0.055 |  | 21.9 (0.9–566.3) | 0.063 |  | 14.6 (0.7–292.6) | 0.080 |  | 27.2 (1.1–648.2) | 0.041 |
| Reproductive and genital tract^†^ |  |  |  |  |  |  |  |  |  |  |  |
| Breast | 1.2 (0.7–1.9) | 0.568 |  | 2.0 (1.1–3.7) | 0.023 |  | 2.0 (1.2–3.4) | 0.013 |  | 2.7 (1.5–4.9) | 0.001 |
| Uterine cervix | 0.2 (0.0–1.7) | 0.146 |  | 0.5 (0.1–4.3) | 0.508 |  | 0.5 (0.2–1.4) | 0.199 |  | 0.7 (0.3–1.8) | 0.451 |
| Uterus | 1.5 (0.4–6.6) | 0.572 |  | 4.7 (0.9–24.3) | 0.064 |  | 3.9 (0.8–18.3) | 0.080 |  | 4.4 (0.9–21.3) | 0.068 |
| Ovary | 0.6 (0.2–1.7) | 0.363 |  | 1.2 (0.4–4.0) | 0.725 |  | 1.6 (0.5–5.1) | 0.439 |  | 2.3 (0.7–8.2) | 0.185 |
| Vulva, vagina | NA | NA |  | NA | NA |  | 0.2 (0.0–10.4) | 0.453 |  | 0.1 (0.0–7.6) | 0.340 |
| Prostate | 1.0 (0.4–2.6) | 0.955 |  | 0.4 (0.2–1.3) | 0.145 |  | 1.1 (0.7–1.6) | 0.678 |  | 0.8 (0.5–1.3) | 0.355 |
| Testis | 4.3 (0.0–391.1) | 0.525 |  | 0.9 (0.0–120.0) | 0.962 |  | 1.8 (0.2–15.2) | 0.596 |  | 2.0 (0.2–18.8) | 0.554 |
| Urinary tract |  |  |  |  |  |  |  |  |  |  |  |
| Kidney | 23.9 (8.6–66.5) | < 0.001 |  | 20.0 (7.0–57.3) | < 0.001 |  | 10.4 (4.8–22.7) | < 0.001 |  | 11.1 (5.0–24.6) | < 0.001 |
| Bladder/urinary tract | 4.3 (1.6–11.6) | 0.004 |  | 4.0 (1.3–11.9) | 0.013 |  | 3.9 (2.0–7.4) | < 0.001 |  | 3.8 (1.9–7.7) | < 0.001 |
| Brain | 2.2 (0.3–15.9) | 0.434 |  | 1.8 (0.2–15.9) | 0.619 |  | 0.6 (0.2–1.7) | 0.357 |  | 0.9 (0.3–2.5) | 0.891 |
| Thyroid | 1.4 (1.0–2.0) | 0.076 |  | 2.0 (1.3–3.0) | 0.001 |  | 1.8 (1.2–2.6) | 0.003 |  | 2.0 (1.3–3.0) | 0.001 |
| Hematopoietic system |  |  |  |  |  |  |  |  |  |  |  |
| Hodgkin’s lymphoma | 5.5 (0.1–519.7) | 0.461 |  | 295.7 (0.1–699830.4) | 0.151 |  | 0.6 (0.1–6.8) | 0.708 |  | 0.6 (0.1–7.6) | 0.730 |
| Non–Hodgkin’s lymphoma | 3.6 (2.0–6.5) | < 0.001 |  | 8.1 (4.1–16.0) | < 0.001 |  | 3.0 (1.7–5.4) | < 0.001 |  | 3.2 (1.7–5.9) | < 0.001 |
| Multiple myeloma | 10.9 (0.3–465.4) | 0.212 |  | 7.2 (0.1–392.0) | 0.332 |  | 1.5 (0.4–5.2) | 0.520 |  | 0.8 (0.2–3.3) | 0.791 |
| Leukemia | 0.4 (0.0–3.1) | 0.367 |  | 0.2 (0.0–2.1) | 0.194 |  | 1.2 (0.5–3.1) | 0.679 |  | 1.0 (0.3–2.8) | 0.985 |
| Unknown primary | 3.9 (1.3–11.8) | 0.016 |  | 2.5 (0.7–8.8) | 0.157 |  | 1.9 (1.0–3.4) | 0.036 |  | 1.9 (1.0–3.6) | 0.054 |
| Total | 1.9 (1.6–2.3) | < 0.001 |  | 2.0 (1.7–2.4) | < 0.001 |  | 1.6 (1.5–1.8) | < 0.001 |  | 1.6 (1.4–1.8) | < 0.001 |

*HR* hazard ratio, *CI* confidence interval, *NA* not applicable.

The relative risk was obtained from the Cox model adjusted for age, sex, and the presence or absence of any of investigated comorbidities as in Table 1.

^*^Non-melanoma skin cancer is a composite of Merkel, squamous, and basal cell carcinomas.
^†^Cancers of the breast and female genital tracts were investigated only in females, whereas prostate and testicular cancers were analyzed only in males.
